# A single *aspergillus fumigatus* intracranial abscess in an immunocompetent patient with parietal lobe tumorectomy

**DOI:** 10.1186/1477-7819-12-181

**Published:** 2014-06-07

**Authors:** Zhao-Shi Bao, Gan You, Wen-Bin Li, Tao Jiang

**Affiliations:** 1 Beijing Neurosurgical Institute, Beijing 100050, China; 2 Department of Neurosurgery, Beijing Tiantan Hospital, Capital Medical University, Beijing 100050, China; 3 Department of Oncology, Beijing Shijitan Hospital, Beijing, China; 4 Chinese Glioma Cooperative Group (CGCG

**Keywords:** *Aspergillus fumigatus*, Intracranial abscess, Procedure of intracranial infection, Antifungal therapy

## Abstract

Aspergillosis of the central nervous system is a rare fungal infection that is mainly reported in patients with immune deficiency, such as AIDS patients and organ transplant patients treated with immunosuppressive agents, and is uncommon among patients with intact immune function. We report here a rare case of intracranial aspergillosis in a patient who had previously undergone a parietal lobe tumorectomy. *Aspergillus fumigatus* was confirmed by histopathology, and susceptibility tests reported that this infection should respond to voriconazole. We believe the immunosuppression resulting from surgical trauma and glucocorticosteroid treatment may be contributing to the infection, and therefore management of these two factors may improve the prognosis.

## Background

Aspergillosis is an infection of tissues or cavities caused by fungi of the genus *Aspergillus*[1]. Aspergillosis of the central nervous system is a rare disease with a high mortality that occurs more frequently in immunocompromised patients and is not often observed in immunocompetent patients. The most common species to cause aspergillosis of the CNS is *Aspergillus fumigatus*[2]. *A. fumigatus* is found in the environment worldwide, but only a few strains are pathogenic. Finding the source of the infection is difficult, and the treatment of aspergillosis is controversial. It may commonly present as meningitis, fungal granuloma, mycotic aneurysm, or infarction. The prognosis of the CNS aspergillosis, especially intracerebral aspergillosis, is very poor, with a high mortality ranging from 66% to 100% [3].

The case reported here is of an immunocompetent male patient presenting with a simple Aspergillus intracranial abscess 3 months after a craniotomy for a meningioma. He underwent five procedures for the intracranial infection in conjunction with antifungal therapy and achieved a temporary remission. Unfortunately, the patient died of recurrent infection 1.5 years after discharge from our hospital.

## Case presentation

**Figure 1:**
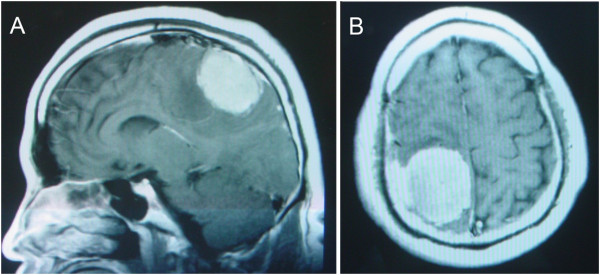
**Images of the patient before the first procedure.** Sagittal **(A)** and axial **(B)** contrast-enhanced MRI scan prior to the first admission demonstrating an enhanced lesion located in the right parietal lobes with the enhanced meninges. (T1W).

A 42-year-old male patient underwent right parietal lobe tumorectomy for a meningioma at Beijing Tiantan Hospital in 2005 (Figure 1). He was discharged 8 days after the surgery with a normal temperature and normal muscle strength but an abnormal hemogram. Three months later, he presented to a local hospital with pus at the surgical incision site and was diagnosed with a surgical site infection. One month later, he underwent a decompressive craniotomy for increased intracranial pressure at the local hospital. He was then transferred to our hospital for better symptomatic management, pus evacuation and treatment for occasional epilepsy. On admission, he was oriented but had white exudates posterior to the incision site. He could move his limbs, and his muscle strength was normal. Computed tomography (CT) revealed enhancement of the dura with the surgical area surrounded by cerebral edema.

### Operation and treatment

The patient was treated with a third generation cephalosporin for 10 days after the evacuation of the yellow-white pus posterior to the incision site via an arche-cut was performed. Three weeks later, he experienced weakness of his left extremities and hyperspasticity of his right extremities. Routine blood tests showed an intermediate cell percentage of 11.8%. CT and magnetic resonance imaging (MRI) revealed an enhanced cavity wall with an apparent brain tissue hydrocephalus.

Three months after the last surgery, the patient underwent another procedure to treat the intracranial infection. The thick abscess wall taken during the operation was identified as an *Aspergillus* granuloma. After the surgery, he was treated with an anti-epileptic and intravenous administration of fluconazole for 1 week. Three weeks later, a broken pustule was found on the incision site and was treated with drainage and flushing with penicillin, gentamicin, and cephalosporin.

**Figure 2:**
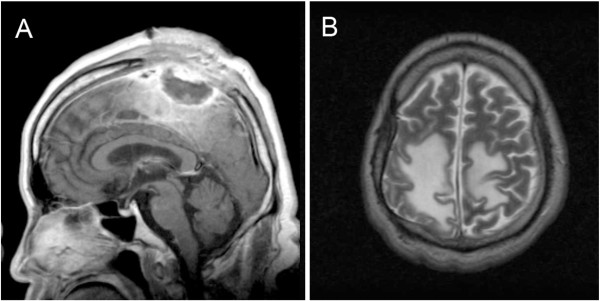
**Images of the patient after the second procedure due to intracranial infection.** Sagittal contrast-enhanced MRI scan **(A)** and axial T2-weighted image **(B)** prior to the third procedure of the infection demonstrating an irregular lesion on the apical lobe with massive hydrocephalus.

**Figure 3:**
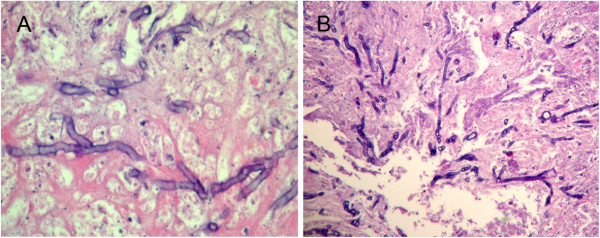
**Pathology of the specimens and secretions of the patient.** Microscopic observation of the fungi demonstrating colonies of septate **(A)** and acute angled, branched **(B)** fungal hyphae. **(A)** Silver staining × 400, **(B)** Silver staining × 200.

After another 2 months, he developed walking dysfunction, along with a diminished muscle strength of 4/5 in his left upper limb and 3/5 in his left lower limb. MRI demonstrated an irregular lesion on the apical lobe, and there was massive hydrocephalus (Figure 2). He then underwent his third and fourth operations for the intracranial infection. The wound was drained and flushed with fluconazole and attenuated iodophors. The presence of *A. fumigatus* was confirmed in the specimens and secretions tested by the Skin & Fungus Lab of Peking University First Hospital (Figure 3). Intravenous administration of voriconazole was started according to the susceptibility test, with 400 mg bid on the first day and 200 mg bid from the second day on. Secretions from the wound decreased.

Sixteen days later, a new open abscessed cavity was found at the front of the wound. A cranial CT scan revealed obvious hydrocephalus that involved the motion area. He underwent a procedure for the intracranial infection with an expansion of the original incision site to resect the hypodermis, the abnormal aponeurosis of the occipitofrontalis muscle and the abnormal brain tissue. During the postsurgical physical examination, his muscle strength of the right limb was 4/5 compared with the 0/5 muscle strength in his left limb. His body temperature was elevated at 38.8°C, and a lumbar puncture was performed that revealed leukocytosis in the collected sample of yellow CSF. Intravenous voriconzole was started, resulting in the cessation of pus secretion from the wound. However, the patient started exhibiting some psychiatric symptoms, such as absentmindedness, apathy, and depression, after 2 weeks of oral voriconazole treatment. He was then treated with intravenous itraconazole. One month later, another CT demonstrated that there was no hydrops in the hypodermis layer. He was discharged when he was afebrile and had a normal tension on the operation area. The muscle strength of his proximal left upper limb was 3/5. His distal left upper limb and left lower limb showed a 0/5 muscle strength. His right upper and lower limbs were 4/5.

This patient died from recurrent infection 1.5 years later. The FBS of this patient was continuously normal during the admission, indicating that the patient was not diabetic. The WBC of this patient was normal and the bacterial culture was negative during the admission.

## Discussion

Aspergillosis of the CNS is associated with a high mortality and is extremely rare in immunocompetent patients [3], especially after open surgery. There are only five reports in the literature of the aspergillosis in the CNS after craniotomy or tumorectomy in the last two decades [2,4-7].

Previous reports indicated that the most common sources of the CNS aspergillosis in immunocompetent patients are the paranasal sinus, the lungs, and the gastrointestinal tract. Intracranial infection occurs more frequently by hematogenous routes and less frequently by direct or contiguous spread [1,8]. *A. fumigatus* conidia are abundant in the air and can be inhaled into the lungs by anyone [9]. They can invade directly into the vessel wall and spread to brain tissue by a hematogenous route from the lungs [1]. In our case, no signs of lung infection were found. His first tumorectomy was located in the right parietal lobe, which was not associated with the base of the skull or the paranasal area. Walsh et al. reported that infection of the maxillary sinus might be complicated by direct invasion into the palate with perforation into the oral cavity or perforation of the nasal septum [10]. However, the infection in this patient was localized to the site of the initial tumor. There was no evidence to prove that *A. fumigatus* was disseminated from the paranasals by direct or contiguous spread in this case.

Although this patient’s immune system was intact, he was given an 8-day course of dexamethasone to treat his hydrocephalus. Glucocorticosteroid decreases the chemotactic and phagocytic function of lymphocytes, which play an important role in immune surveillance. In addition, there may be a state of impaired defense and increased susceptibility to infection and septic complications during the early postoperative period [11]. We believe that the glucocorticosteroid treatment and the postoperative immune suppression may be two potential risk factors for Aspergillus infection of the CNS. Our patient had recurrent disease 1.5 years later. This recurrence suggests an association between infection and reduced immunity. Marinovic et al. reported a case of Aspergillus infection following craniofacial trauma that involved the sinus, and the source of the infection was thought to be the concrete debris [8]. Kim et al. did not find the source of the postsurgical Aspergillus infection in their case [5].

It took 3 months to confirm the diagnosis in our patient due to a negative *A. fumigatus* culture. However, the gold standard for diagnosis of aspergillosis is histopathology. Although specific diagnosis might be possible with some serological tests [5] and Masahiro Kami et al. suggested that DWI and PCR of CSF could be useful in the early diagnosis of CNS aspergillosis [12], the use of these diagnostic techniques has not been fully developed.

Treatment of CNS aspergillosis solely with antifungal agents has produced disappointing results [3]. The most likely reason for the low effectiveness is the poor CNS penetration of antifungal drugs [8], which leaves neurosurgical intervention combined with antifungal therapy as the only acceptable option for treating CNS aspergillosis [4,5,8]. Turgut had discussed a more effective therapy for invasive fungal granuloma due to Aspergillus fumigatus in an immunosuppressed patient [7]. A decompressive craniotomy and five procedures at the intracranial infection site in conjunction with voriconazole and itraconazole therapy were performed on our patient with immunocompetent status to produce a temporary remission.

Voriconazole is the recommended therapy for CNS aspergillosis, while itraconazole and posaconazole have also been successful in treatment of CNS aspergillosis [10]. Prior to publication of Voriconazole recommendation, amphotericin B had been the mainstay of therapy for the past 25 years [1]. Marinovic and Siddiqui reported high Amphotericin B effectiveness in their cases [3,8], while Schwartz et al. concluded that treatment with Amphotericin B and itraconazole was not effective [13]. In our case, the patient was treated by voriconazole for 23 days followed by itraconazole for 23 days, when the infection was controlled.

Our patient died of recurrent infection 1.5 years later, possibly due to a secondary Aspergillus infection that occurred during the prolonged time it took to sort out the original infection.

His relapse and death from the infection 1.5 years later might be related to a progressive immunodeficiency due to the glucocorticosteroid treatment and postoperative immune suppression. We believe that better management of these two factors might have improved the prognosis.

## Conclusions

Aspergillus infection in the CNS is an extremely rare disease. Although the source of the infection after the aseptic craniectomy was not clear, immunosuppression from surgical trauma and glucocorticosteroid treatment may have contributed to the infection. Early diagnostic confirmation of CNS Aspergillosis can improve the treatment response and prognosis. Neurosurgical intervention combined with voriconazole and itraconazole therapy is useful in the treatment of patients with CNS Aspergillosis. Susceptibility testing to confirm the optimal drugs combined with neurosurgery therapy may improve the prognosis of patients with CNS Aspergillosis.

## Consent

Written informed consent was obtained from the patient’s family for publication of this case report and any accompanying images.

## Abbreviations

CNS: Central nervous system; CT: Computed tomography; MRI: Magnetic resonance imaging; CSF: Cerebrospinal fluid; DWI: Diffusion weighted imaging; PCR: Polymerase chain reaction.

## Competing interests

The authors declare that they have no competing interests.

## Authors’ contributions

ZSB participated in the design of the study, sample collection, and drafting of the manuscript. GY performed sample collection. WBL carried out the immunohistochemistry analysis. TJ conceived the study, participated in its design and coordination, and helped draft the manuscript. All authors read and approved the final manuscript.
